# A nanoporous gold membrane for sensing applications

**DOI:** 10.1016/j.sbsr.2016.01.001

**Published:** 2016-03

**Authors:** Swe Zin Oo, Gloria Silva, Francesca Carpignano, Adnane Noual, Katrin Pechstedt, Luis Mateos, James A. Grant-Jacob, Bill Brocklesby, Peter Horak, Martin Charlton, Stuart A. Boden, Tracy Melvin

**Affiliations:** a Optoelectronics Research Centre, University of Southampton, Southampton SO17 1BJ, UK; b Dipartimento di Ingegneria Industriale e dell'Informazione, Università degli Studi di Pavia, Pavia, Italy; c School of Electronics and Computer Science, University of Southampton, Southampton SO17 1BJ, UK

**Keywords:** Nanopore, Polymer sphere, Gold membrane, Plasmons, Sensing, SERS

## Abstract

Design and fabrication of three-dimensionally structured, gold membranes containing hexagonally close-packed microcavities with nanopores in the base, are described. Our aim is to create a nanoporous structure with localized enhancement of the fluorescence or Raman scattering at, and in the nanopore when excited with light of approximately 600 nm, with a view to provide sensitive detection of biomolecules. A range of geometries of the nanopore integrated into hexagonally close-packed assemblies of gold micro-cavities was first evaluated theoretically. The optimal size and shape of the nanopore in a single microcavity were then considered to provide the highest localized plasmon enhancement (of fluorescence or Raman scattering) at the very center of the nanopore for a bioanalyte traversing through. The optimized design was established to be a 1200 nm diameter cavity of 600 nm depth with a 50 nm square nanopore with rounded corners in the base. A gold 3D-structured membrane containing these sized microcavities with the integrated nanopore was successfully fabricated and ‘proof of concept’ Raman scattering experiments are described.

## Highlights

• 3D structured gold microcavity membrane with a nanopore in the base designed
• Theoretically for plasmon-enhanced detection
• Fabrication of gold microcavity membrane with a nanopore
• ‘Proof of concept’ study showing enhanced Raman scattering detection

## Graphical abstract

**Figure:**
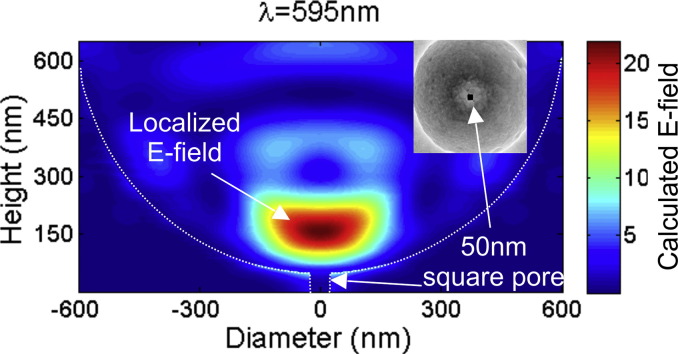

## 1 Introduction

Biosensors for the direct detection of biomolecules at the single molecule level would be highly desirable for a range of diagnostic applications, especially if these bioanalytes could be delivered sequentially through a nanopore. So far, metallic nanostructures have been shown to provide an enhancement factor of 10^14^ with a large cross-sectional area of 10^− 16^ cm^2^/molecule using surface enhanced Raman scattering approaches [1]. The rational design of metallic nanostructures for this application, also known as plasmonic substrates or devices, for sensing applications is well established [2]; the functional characteristics of these devices are based upon the behaviour of plasmons at the interface of a metal-dielectric medium. For metallic nanostructures, the electrons in the metal are excited and oscillate within the metal core near the interface with a surrounding dielectric material; these collective electron excitations are known as surface plasmon polaritons.

The potential of nanostructured metallic structures for optical applications has been demonstrated for (i) biosensing applications [3], (ii) surface enhanced Raman spectroscopy (SERS) [4], (iii) guiding and manipulating the light [5], (iv) sub-diffraction limited imaging [6] and (v) trapping of micro/nano-sized particles [7]. In order to apply the nanostructures for these applications, the structure dimensions and geometry have to be appropriate for (i) tuning of the plasmon resonance coupling, (ii) the near field enhancement, (iii) the confinement in sub-wavelength region coupling, (iv) enhanced evanescent waves and (v) the near- and far-field enhancement, [8] respectively to each application. Moreover it is essential that there are high levels of local-electric field intensity at locations within the sensor where the plasmon enhancement of signal from the transducer will be most beneficial — this is especially true for sensing applications where the analyte must be within the region of highest electric field (*E*-field) intensity. Various metallic nanostructures have been proposed for sensing using a range of different shapes and geometries [9], materials [10], [11] and fabrication methods [12], notably for the optimization of SERS measurements [13] tip-enhanced Raman scattering, (TERS) [14] and fluorescent enhancement [15]. Most of the nanostructures studied so far are multi-scale nanoparticles and nanocrystals. The nanoparticles can be classified as 1-dimensional [16], 2-dimensional and 3-dimensional nanoparticles i.e., nanorods, nanocrescent and nanopores respectively. More recently planar plasmonic substrates have been developed for ‘nanofocusing of plasmons’ [17], [18], [19], [20], [21], [22] and SERS [23], [24].

Although TERS has been applied for the sequence analysis of DNA [25], such a measurement is highly challenging from a practical point of view. Whether nanopores could be designed to yield plasmonic sensors appropriate for the direct detection of single molecules within the pore is a question that still remains. In other words, could the *E*-field be highly confined within the nanopore with a view to providing a sufficiently sensitive nanopore sensor for single molecule detection at translocation?

Various groups have studied the plasmonic properties of 2-dimensional nanoporous gold films where a change in the pore size provides a shift in the wavelength of the band assign to plasmon resonance [26], [27]. Unfortunately, so far, the level of plasmon enhancement is not reproducible which is possibly due to a variation in pore shape and size of the fabricated devices [28], [29], [30], [31], [32]. Various teams have studied plasmonic devices based upon the so-called ‘bowtie’ configuration [31], [32], [33]. These structures consist of sharp triangular metallic structures in the shape of a bowtie, where the distance between the two closest triangle tips (the bowtie antennas) can be used to tune the plasmon resonance. Where there is a nano-sized pore located between the bowtie antennas, the localized *E*-field is confined at the pore, in between these antennas [33], yielding a 2D-structured metallic device with an integrated pore. So far the only example of precise 3D-structured metallic substrates with pores have been reported by Lindquist et al., where the 3D nanopore device is a hybrid of metallic pyramid with C-shaped apertures to tailor the plasmonic properties for the application of TERS [29].

In this study we report the design, fabrication and first evaluation results of a 3D-structured nanoporous structure where the *E*-field intensity is highly localized ‘at’ and ‘inside’ the pore for sensitive biosensing applications where the analyte is to be passed through the pore.

## 2 Materials and methods

### 2.1 Theoretical design of microcavity with integrated nanopore

**Fig. 1:**
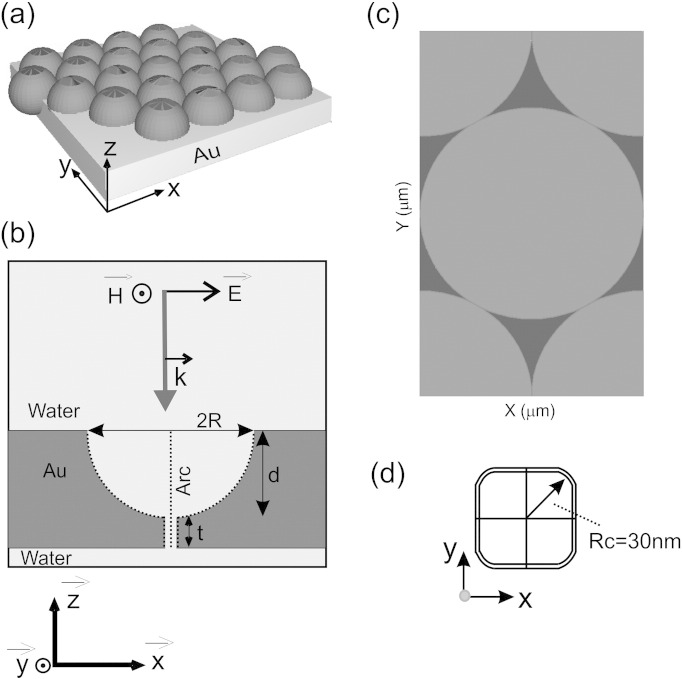
(a) 3-Dimensional view of the gold nanoporous micro-cavities used for the theoretical simulations in which spheres were arranged in the hexagonal lattice and the height of the sphere immersed in the gold film (grey block) was set to 50% of the diameter of sphere. The background medium was defined as water or air. (b) 2-Dimensional cross-sectional view of the nanoporous microcavity plus the incoming EM field (The dotted line labelled arc is used for ascending plots of *E*-field intensity as described in the text.) (c) The top view (XY-plane) of the gold membrane which shows a single unit cell of the hexagonal lattice (1200 nm diameter micro-cavity). (d) The top view of the pore upper cross section where R_c_ is defined as the radius from the centre of the square pore.

Fig. 1(b) contains a schematic of the cross-sectional structure used for the theoretical studies. The model consists of a thin layer of water (or air) on which the three-dimensionally structured gold microcavity with a nanopore is placed, in and above the microcavity and the pore. The surrounding medium is also water (or air). As the model in this first instance is a single microcavity, the scattering boundary condition (the scattered field is considered to be fully absorbed at the boundaries of the system, the boundary is transparent to the scattered and the incident *E*-field) is applied outside the structure shown in Fig. 1(b). The cavity radius (R) is varied for the initial studies, and the depth of the cavity (d) is set to R. Several simulation approaches are applied:

*Method 1*: Three-dimensional hexagonal close-packed micro-cavities were considered theoretically using the software suite, RSoft DiffractMOD, Synopsis Inc. DiffractMOD employs the Rigorous Coupled Wave Analysis (RCWA) method to obtain the backward diffraction efficiency; periodic boundary conditions are used, as previously described for a different system [34].

In brief, the number of harmonics is set to 5 for the in-plane surface to expand the refractive index and field in Fourier space. The excitation light, which is normal to the opening of the porous membrane, is a plane wave with p-polarized light. The simulations are performed for a wide spectral range (400 nm to 950 nm with 2.5 nm steps), typically with p-polarization. As a figure of merit, the zero order diffraction efficiency (specular reflection) is also obtained. In order to properly establish the *E*-field in and around the drilled square hole, the spatial *E*-averaged power monitor (*U*_*E*_) given by the Eq. 1 below, is applied:

(1) UE=12∫VRe∈rEr2dV.

Where E(r) is the *E*-field, ∈(r) is the spatially dependent permittivity and the integration is performed over V, which is the volume of monitor set by length and height/width. The index resolution of the field monitor is set to 1 nm within the nanopore region. The mesh size is 1 nm. The model geometry used to simulate the microcavity film to be fabricated, is shown in Fig. 1 where the base gold thickness is 150 nm, the depth of the cavity is 50% of the diameter of the sphere and the nanopore is 50 nm × 50 nm square. Each unit cell, as shown in Fig. 1(c), is treated as a periodic array and the transmission line formulation for the boundary condition and the z-direction is defined as the launch field.

*Method 2*: The Comsol-Multiphysics Package (Finite-Element-Method) was used to determine the *E*-field localization, distribution and scattering of a single microcavity. For simplicity, even though a full-3D simulation could be done, only one quarter of the structure needs to be simulated because the incoming plane wave (up on normal incidence) is linearly polarized, and as so the total system (geometry and perpendicular incoming wave) contains two symmetry planes [35]. When the incoming field is at an oblique incidence angle, the symmetry is broken so, in this case a full-3D model is required to include the oblique incident light. The underpinning theory and method are described in full detail in the supplementary information (S.I.), along with the model validation results compared with analytical data produced using another published method [36].

### 2.2 Fabrication of the 3D-structured gold nanoporous membrane

**Fig. 2:**
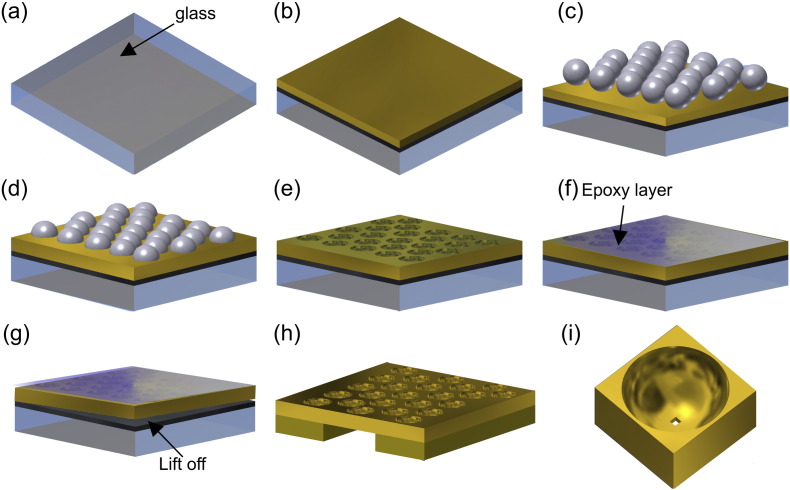
Schematic showing the fabrication steps used to create the 3D-structured gold membrane with nanopores. (a–c) Self-assembly of 1.2 μm diameter polymer spheres onto a Teflon and then gold-sputtered glass substrate. (d) The self-assembled spheres provide a template for subsequent electroplating of gold to achieve the required 600 nm cavity depth. (e) The polymer spheres were dissolved away in dimethylformamide. (f) Epoxy coating on the sample. (g) The gold membrane was released from the Teflon-coated ‘handle’ substrate by lift off. (h) This yields a gold membrane, supported on a gold TEM (reference) grid that is subsequently milled in an ORION Plus Helium Ion Microscope (Carl Zeiss). (i) The 50 nm square nanopore at the base of the micro-cavity.

The fabrication process steps are shown schematically in Fig. 2. The cleaned glass substrate was coated with 50 nm of Teflon with a deposition rate of 0.07–0.26 Å/s using the resistance evaporator (BOC Edwards Auto 360). This was followed by gold deposition with the e-beam evaporator (BOC Edwards) with a deposition rate of 0.03 Å/s. Self-assembly of polymer spheres onto the gold thin film was used. The mono-disperse microspheres used in this study are the carboxylate modified latex polymer spheres, 4%*w*/*v*, 1.2 μm dia. (Invitrogen). Before the deposition of the polymer spheres, one end of the gold surface was covered with parafilm for later use in electroplating as an anode. The parafilm was adhered by heating on a hot plate at 40 °C. The 1 wt.% solution was freshly prepared in deionized water and diluted with ethanol to 50% and assembled on the surface as previously described [37]. Once the sample was dry, the parafilm was removed by heating at 40 °C for ~ 2 min. For electroplating around the polymer spheres the non-cyanide gold solution (ECF-60) from Metalor Technologies was used and Brightener E3 (Metalor) was added to the gold solution at a ratio of 1:75. Gold electrodeposition was then achieved at room temperature using a three-electrode configuration controlled by Autolab PGSTAT12 under potentiostatic condition at − 0.615 V versus control electrode. (For full details of electrodeposition of gold with the self-assembled polymer spheres, see S.I. for description and figures/images of fabrication (Figures S7–S11). As soon as the sample had been electroplated, it was immersed in dimethylformamide (Sigma-Aldrich) to remove the polymer spheres (for > 15 min).

The gold structured film was then coated in epoxy and carefully lifted off from the Teflon surface using a razor blade. This yielded free-standing, gold microcavity films which were then placed on top of a gold TEM grid. The TEM grid with the fabricated gold structure film (with the epoxy on the top side) was placed within some modified tweezers (see Figure S7, S.I.). The tweezer-held 3D-structured gold membrane/TEM grid was placed into acetone and the epoxy carefully dissolved away without releasing the membrane from the tweezers. Milling of the nanopore in the base was performed using an ORION Plus Helium Ion Microscope, HIM (Carl Zeiss). This produces a focused beam of helium ions with a sub-nm probe size that can be used for controlled, highly-localized ablation of material in a similar way to conventional gallium ion FIB but with a higher degree of precision [38]. Nanopore milling was performed by raster scanning of the focused He ion beam over the area to be milled using the following parameters: a beam energy of 30 keV, a beam current of 0.5–1 pA, a working distance of 8.5 mm, a raster scan and a dwell time per pixel of 3-10 μs. The beam was first focused on the base of the cavity in imaging mode and any beam astigmatism was corrected. The field of view was then reduced to an area of 25 nm × 25 nm and the beam was raster scanned, milling away material in this area to form an initial hole. The secondary electron emission was monitored to determine the end point of milling (complete penetration to other side of thin film). The field of view was then increased to 50 nm × 50 nm and centred on the milled 25 nm hole. The beam was then raster scanned to complete the pore formation; the expansion of the hole to fill the 50 nm × 50 nm square area was monitored in secondary electron imaging mode.

### 2.3 ‘Proof of concept’ Raman spectroscopy evaluation of the nanoporous gold membrane

The microcavity containing milled nanopores located relative to identifiers in the TEM grid was coated with a drop of 5 mM benzenethiol (Sigma Aldrich (assay ≥ 99%) in absolute ethanol for 24 h. The sample was thoroughly rinsed in absolute ethanol and dried. Raman measurement was carried out using a Renishaw InVia Raman Spectrometer system at 633 nm excitation wavelength, 0.5 mW power using 100 times magnification with numerical aperture 0.8. The sample was set at 1 mm working distance and highly confocal imaging using a 20 μm diameter pinhole, was applied.

## 3 Results and discussion

### 3.1 Design of the microcavity with a nanopore

**Fig. 3:**
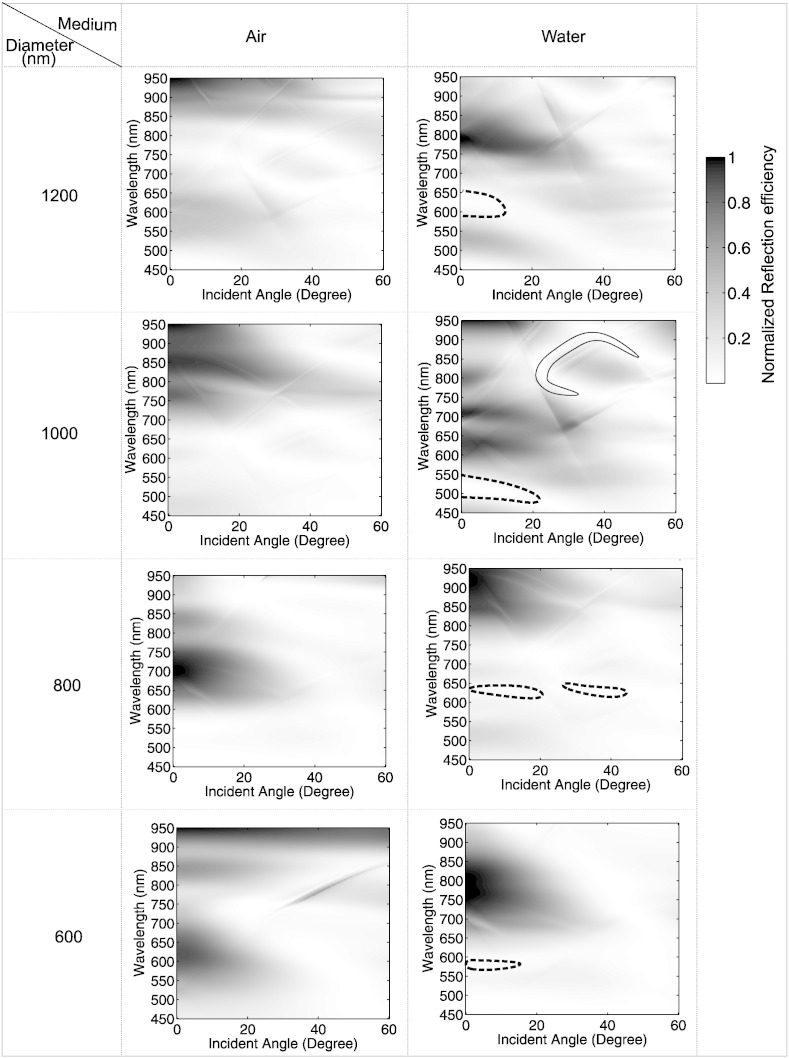
Maps of normalized reflection as a function of wavelength with the incident angle for the different diameters of micro-cavity under an air or water environment.

Micro-cavities (with no nanopore) of the configuration shown in Fig. 1(a) with diameters of 1200 nm, 1000 nm, 800 nm and 600 nm and with depths of half the diameter were evaluated theoretically (using the RSoft DiffractMOD software) to obtain dispersion maps. Surface plasmons are said to be delocalized (propagating) surface plasmon polaritons if they travel along the interface of the metal and dielectric medium at the top surface of the substrate. They can be excited via scattering as a result of the periodicity of the structure and have sharp (narrowband) absorption properties. The dispersion maps, shown in Fig. 3, are formed by mapping reflection efficiency vs. wavelength as a function of the illuminated incident angles (0° to 60°) and they were simulated for hexagonal closed-packed micro-cavity structures immersed in air or water. The reduction in the reflection efficiency is assumed to be the result of the plasmon induced absorption. The plasmon energies are highly dependent on the incident angle and lattice orientation of the microcavities (notably for propagating plasmons). As previously described, the reflectivity plots can be used to extract features characteristic for the identification of non-dispersive localized and propagating plasmons [39]. Plasmon ‘bands’ assigned to localized plasmons are broader than those for the propagating plasmons and their plasmon energies are insensitive to the incident angles. Surface plasmons localized within the cavity such that the *E*-field is confined inside the microcavity are known as localized plasmons.

From the plots of the reflection efficiency versus wavelength obtained for the microcavities in air (Fig. 3 first column), it can be seen that as the micro-cavity diameter decreases there is a blue shift of the absorption in the map, assigned to the localized plasmon resonance. It is more difficult to observe a trend in the data obtained for micro-cavities in the water medium (Fig. 3, second column). This is because there is a dramatic difference between the dispersion maps obtained for the 1000 nm and 800 nm diameter micro-cavities. Regions are identified in the maps as examples of localized plasmons (surrounded by the black dotted lines); these regions are where the reflection is lowest and which does not vary significantly with the incident angle. An example of the conditions for obtaining propagating plasmons, is identified by the surrounding black line; these show a strong variation with incident illumination angle. The examples are extracted from a contour form of the normalized reflection efficiency map (not shown). The different refractive index values for the medium (water and air) clearly have a marked effect on plasmon tuning. It is obvious that changing the size of the gold micro-cavity can be used to selectively tune the plasmon resonance frequency. For biosensing, water is the most common medium used. The plasmon resonance wavelength is tuned for approximately 600 nm and 650 nm for the 600 nm and 800 nm diameters of the micro-cavity, respectively. When the diameter of the cavity is larger, the localized plasmon regions of the dispersion map, as indicated by the dotted lines, occur over broader wavelength regions: 500 nm–550 nm and 595 nm–655 nm band for the 1000 nm and 1200 nm cavity diameters, respectively. These shorter wavelengths offer better device functionally for both plasmon-enhanced fluorescence and Raman scattering studies.

The next parameter to be considered is the geometry of the nanopore to be milled in the base of the microcavity. For these studies a single microcavity is considered in the simulation, where the Finite Element Method (FEM) approach is applied (validation of this method is fully described in the S.I. p1–3). Then the *E*-field intensity in a 50 nm pore in a gold film was considered to provide a reference in comparison to pores in the base of the 3D-structured microcavity (see Figures S2 and S3) to compare differences in *E*-field intensity and distribution within the pore. Including the 50 nm square nanopore in the base of a microcavity (1.2 μm diameter, 0.6 μm deep) resulted in a marked increase of the *E*-field intensity (normalized against the input *E*-field of the light) in the base of the microcavity in the nanopore centre and at the nanopore edges (Figure S5, S.I).

**Fig. 4:**
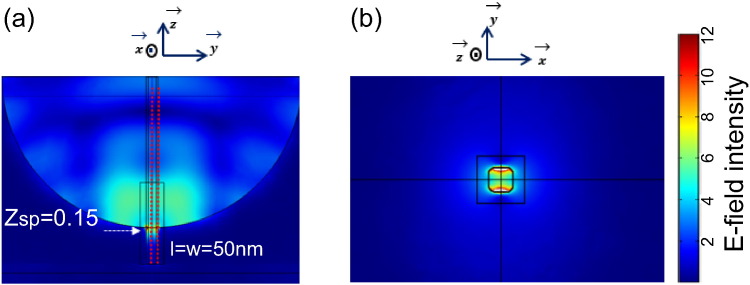
(a) Calculated *E*-field intensity (normalized against the input light *E*-field) for the cross-section of the micro-cavity with 50 nm square nanopore with rounded corners (R_c_ = 30 nm, microcavity diameter = 1.2 μm, λ = 595 nm, P—polarized incident light). The red dashed lines in the centre are the arc along which the normalized *E*-field intensity is plotted (S.I. Figure S5). (b) Normalized *E*-field intensity for the nanopore with rounded corners (R_c_ = 30 nm) at the opening in the base of the microcavity. The *E*-field energy scale bar is for both figures shown.

Although a variety of pores of different dimensions and geometries were considered, our simulations for a 1.2 μm diameter and 0.6 μm deep gold microcavity in water revealed that a pore in the base of 50 nm square with rounded corners (R_c_) of 30 nm radius from the centre of the pore (see Fig. 1(d) for pore design) yielded a device was the optimal configuration evaluated; for this configuration there was a moderate improvement of *E*-field intensity at the centre of the pore, as shown in Fig. 4. This is the point where the analytes are anticipated to travel (by electrophoretic flow for instance), even though the *E*-field at the walls of the pore is more localized and of higher intensity. The advantage of using a square pore with rounded corners is that the *E*-field intensity extends into the centre of the pore, as compared to a circular (data not shown), we also hypothesised that the fabrication of such a pore would be more straightforward than a pore with sharp, squared corners.

**Fig. 5:**
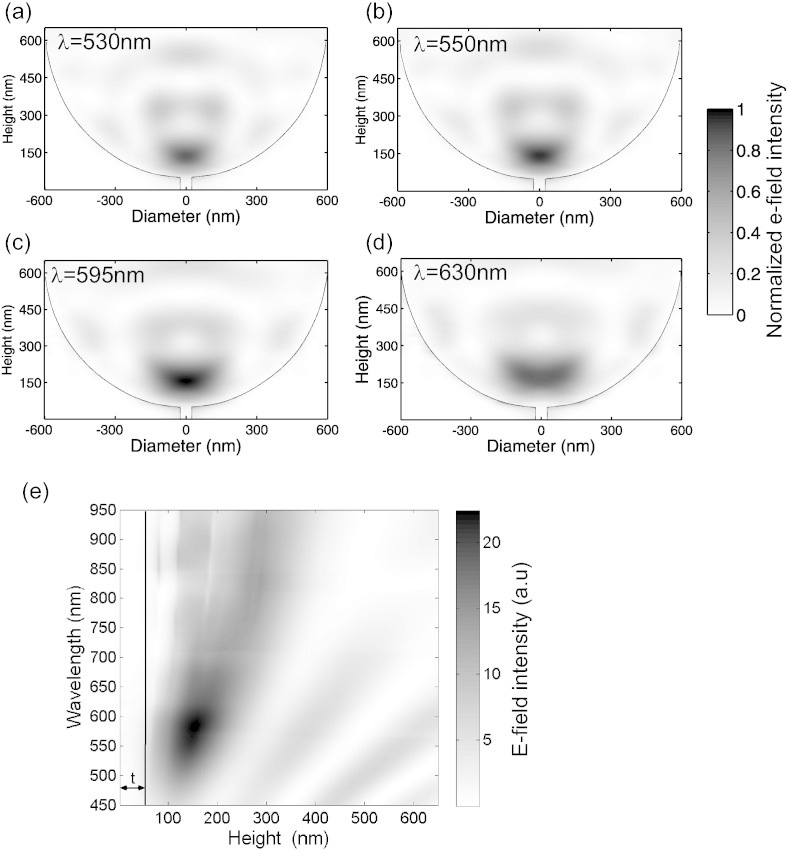
Calculated normalized *E*-field intensity within a 3D micro-cavity (cross-section) with a diameter of 1200 nm and the depth of 600 nm for an incident wavelength of (a) λ = 530 nm, (b) λ = 550 nm, (c) λ = 595 nm and (d) λ = 630 nm. (e) Field intensity along the perpendicular to the centre of the arc for various excitation wavelengths. ‘t’ indicates the nanopore depth as shown in Fig. 1(b).

We thus incorporated a 50 nm nanopore into the base of a 1.2 μm diameter, 0.6 μm microcavity for our further simulations of hexagonal close-packed microcavities. Although the cross-sectional profile of the normalized *E*-field intensity in the cavity can be seen in the data presented in Fig. 4(a), simulations were also performed where the periodic nature of the hexagonally close-packed microcavities is considered (using the RSoft DiffractMOD software). Plots of the normalized*E*-field intensity across the cross section of the microcavity with the 50 nm pore are shown in Fig. 5(a–d) over the wavelength range 530–630 nm. For all cases the highest *E*-field is almost exclusively in the lower part of the microcavity. Supplementary data in the form of a video (Video 1.mp4) provides similar data for a larger spectral range (450–950 nm with 5 nm steps) whereas data for only 4 wavelengths are shown in Fig. 5. The confined *E*-field at the base of the microcavity broadens with an increase in wavelength till the near infrared regime and disappeared in the infrared region (seen in Video 1.mp4). When illustrated with 530 nm, 550 nm, 595 nm and 630 nm (Fig. 5) the highest *E*-field is confined at above the top of the pore. Thus we envisage an analyte to be delivered through the pore in the base of the cavity and to travel, in the majority directly towards the centre of the opening. Thus a map of the *E*-field intensity as a function of wavelength and position from the bottom and centre of the lower nanopore opening shown in Fig. 5(a–d) up to the central position of the microcavity to approximately 100 nm above the top of the device, is shown in Fig. 5(e). The *E*-field intensity along this trajectory varies with the excitation wavelength. The intensity is greatest at the shorter wavelengths and the *E*-field confinement is reduced and dispersed from the nanopore with the longer excitation wavelengths; the ideal operation wavelengths are between ~ 510–650 nm, when the *E*-field intensity is just above the nanopore opening. An excitation wavelength of 595 nm excitation is selected as the operation wavelength with the 1200 nm diameter micro-cavity with 50 nm square nanopore for the application of fluorescent enhancement and SERS. The field intensity map for the 1000 nm diameter cavity as shown in S.I. (Figure S6) shows almost the same distribution as the 1200 nm diameter cavity apart from the field intensity. Therefore the comparison of field intensity at the 595 nm operation wavelength between those two diameter cavities was carried out (seen in Figure S6(b), S.I.). The field intensity of the 1000 nm diameter cavity is 25% lower than that of 1200 nm diameter cavity.

**Fig. 6:**
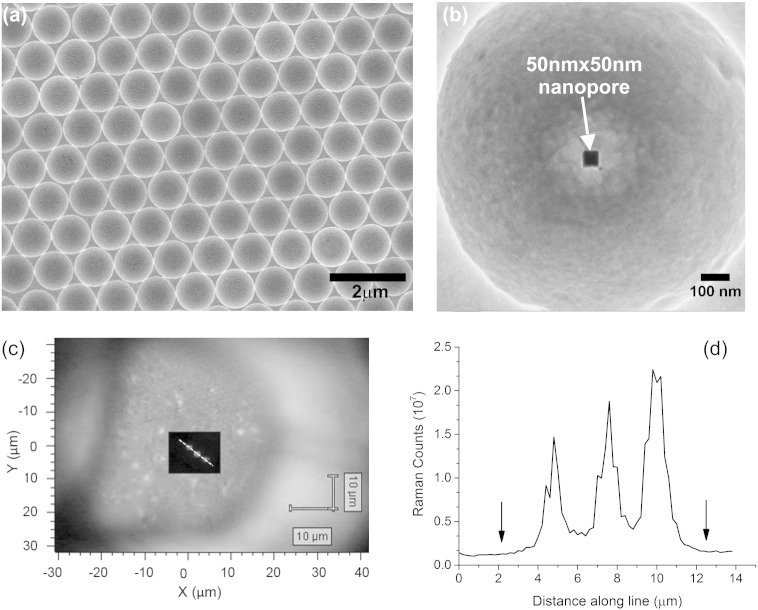
(a) Scanning electron microscope (SEM) image of the electroplated gold micro-cavities on the membrane. The scale bar is 2 μm. (b) Helium ion microscope (HIM) image of a single micro-cavity with a nanopore. The scale bar is 100 nm. (c) Raman spectroscopy of the three-dimensional gold film with three 50 nm square nanopores ~ 2.4 μm apart, after treatment with thiophenol (633 nm excitation wavelength, × 100 magnification) the overlay is a map of the Raman signal intensity (scale black to white) in the area scanned is provided as an overlapping plot over the bright field image of the gold microcavity film with a TEM grid. (d) Plot of the Raman counts (integrated over the whole spectrum measured) with distance for the points indicated by the white line shown in Fig. (c). The arrows on the plot indicate the location along the line where there are the centres of microcavities with no pores.

The gold membrane was fabricated as detailed in Section 2 and shown in Fig. 2. The S.I. (p6–10) contains further details of the fabrication optimization conditions, including the potentiostatic conditions required to achieve gold deposition that conforms to the surface of the self-assembled polymer beads. Following removal of the beads, a SEM image of the microcavity was obtained and is shown in Fig. 6(a). Fig. 6(b) illustrates the HIM image showing the milled square nanopore (50 nm × 50 nm). The corner rounding of square was characterized by the radius of curvature at the four corners and averaged, and was approximately 5.91 nm. It can be seen that the microcavities have relatively smooth surfaces and that the nanopore is centrally located at the base.

Three nanopores (square 50 nm) were milled using the HIM, approximately 2.4 μm apart in the centre at the base of alternate microcavities. In Fig. 6(c), a bright field image of the microcavities within the letter D of the TEM grid is overlaid with a high-resolution scan (500 nm steps) over the microcavity-region where the scan was performed. The highest integrated Raman intensity (white) was observed at the three places where the nanopore exists. The Raman intensity scanned region shown in Fig. 6(c) is the total integrated Raman counts over the entire Raman spectrum. A white dotted line is plotted across the points where the Raman intensity is greatest and is shown in Fig. 6(c), this traversed the hexagonal close-packed cavities with three pores that were known to be at alternate microcavities. Fig. 6(d) plots the line graph of the total integrated Raman counts along the scan line. Again the high Raman count was clearly observed at only the three places where the three pores had been milled, (approximately 2.4 μm apart). This is consistent with what was observed by SEM for the milled pores (data are not shown here). The separation between the peaks is not uniform because the 1.2 μm diameter cavies are not exactly closed-packed. However it can be concluded that the *E*-field is confined at the nanopore resulting in the enhanced Raman scattering and that the signal at the pores in these early ‘proof of concept’ devices is greater for the cavities with a nanopore than for the cavities without nanopores. This large difference between the microcavities without pores and the microcavities with nanopores was not fully expected; it is clear that further studies are required to provide more precisely fabricated devices, however the fabrication of 3D-structured microcavity films in gold with precisely milled nanopore holes suitable for suspending over structures such as TEM grids, is a first. Our theoretical data is a good approximation to the experimental findings of the microcavity nanoporous membranes that are fabricated.

## 4 Conclusions

After a variety of theoretical simulations, the optimal gold microcavity of 1.2 μm with a 50 nm square nanopore with rounded corners, was established. The fabrication of a gold 3D-structured nanoporous membrane provided a structure with relatively good agreement to the optimal theoretical design. This is the first report of this device that is developed for biosensing, notably with a view to yield a system for very sensitive detection of molecules traversing through the nanopore. Preliminary testing of this device provides evidence that there is an enhancement of the Raman signal for thiol molecules attached over the gold surface for the microcavities with nanopores. Our efforts are continuing in the optimization of the device and further testing to provide clearer experimental evidence for plasmon coupling by the nanopore with the micro-cavity.

The following are the supplementary data related to this article.

Video 1 (Video1.mp4) The calculated *E*-field intensity over the cross-section of the nanoporous micro-cavity for different excitation wavelengths. The cross-section was taken along the XZ plane at the center of the gold membrane, as shown in Fig. 1(b) in the paper. The nanopore shape was fixed to 50 nm × 50 nm square and the diameter of sphere was 1200 nm and the height of the cavity was 600 nm. The localized *E*-field was highly confined at just above the nanopore in the fluorescent regime 450 nm–700 nm wavelengths. The *E*-field spreads along the curve of the cavity with up to 650 nm excitation wavelength, and gradually disperses over the cavity in the near infrared regime.

Supplementary Information (S.I.) Contains simulation validation, further simulation data, and further details of the fabrication protocols used to create the 3D structured gold membrane.
