# Supplementary figures and images for: A nanoporous gold membrane for sensing applications
Source: Sens Biosensing Res. 2016 Mar;7:133–40. doi: 10.1016/j.sbsr.2016.01.001 (PMC4783582; doi:10.1016/j.sbsr.2016.01.001)

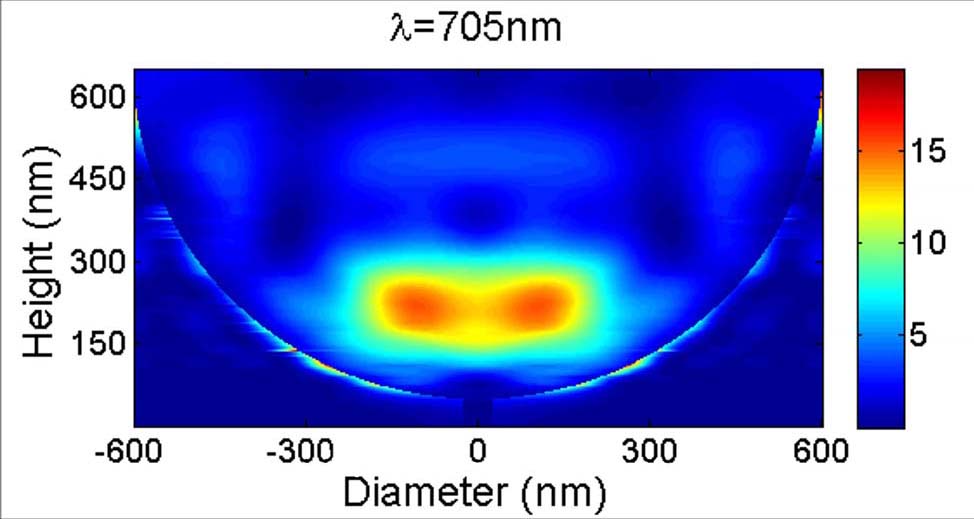

Supplement: Video 1 — (Video1.mp4) The calculated E-field intensity over the cross-section of the nanoporous micro-cavity for different excitation wavelengths. The cross-section was taken along the XZ plane at the center of the gold membrane, as shown in Fig. 1(b) in the paper. The nanopore shape was fixed to 50 nm × 50 nm square and the diameter of sphere was 1200 nm and the height of the cavity was 600 nm. The localized E-field was highly confined at just above the nanopore in the fluorescent regime 450 nm–700 nm wavelengths. The E-field spreads along the curve of the cavity with up to 650 nm excitation wavelength, and gradually disperses over the cavity in the near infrared regime. [file mmc1.jpg]
