# Supplementary material for: A nanoporous gold membrane for sensing applications
Source: Sens Biosensing Res. 2016 Mar;7:133–40. doi: 10.1016/j.sbsr.2016.01.001 (PMC4783582; doi:10.1016/j.sbsr.2016.01.001)
Supplement: Supplementary Information (S.I.) — Contains simulation validation, further simulation data, and further details of the fabrication protocols used to create the 3D structured gold membrane. [file mmc2.pdf]

A nanoporous gold membrane for sensing applications

Swe Zin Oo[1], Gloria Silva[1,2], Francesca Carpignano[1,2], Adnane Noual,[1] Katrin Pechstedt[1], Luis Mateos[1], James A Grant-Jacob[1], Bill Brocklesby[1], Peter Horak[1], Martin Charlton[3], Stuart A Boden[3], Tracy Melvin[1,3]

[1]Optoelectronics Research Centre, University of Southampton, Southampton, SO17 1BJ, UK

[2]Dipartimento di Ingegneria Industriale e dell'Informazione, Università degli Studi di Pavia, Pavia, Italia

[3]School of Electronics and Computer Science, University of Southampton, Southampton, SO17 1BJ, UK

Numerical simulation methods and supplementary theoretical data: The interaction between the EM field and the quasi-free electrons of gold metal is described within the classical framework of Maxwell equations. This can be simply justified by the huge number of conduction electrons in the metal contributing to the coupling with the incident field. By assuming no excess charges and no currents within the simulation domain and a harmonically oscillating ($\vec{E}$ and $\vec{H}$) EM fields, one obtains the (vector) wave equation for the electric field (or similarly for the magnetic field):

$$\nabla\times\left(\frac{1}{\mu}\times\vec{E}\right)-k_o^2\varepsilon\vec{E}=0 \qquad \text{eqn1}$$

where k_0 is the wave number in vacuum, ε and μ are the relative permittivity and relative permeability, respectively, and $\vec{E}$ is the electric field of the incoming wave. The Radio-Frequency module is used (in Comsol) to solve the equation (eqn1). This module is specifically dedicated to electromagnetic problems. Due to the fact that in our system we have only one homogeneous medium (water) plus the metallic cavity, we solved for the scattered field only. For scattering problems the total field can be written as:

$$\vec{E}_t=\vec{E}_b+\vec{E}_s$$

$\vec{E}_b$ is the background field (as named in Comsol), physically, this vector quantity stands for the (oscillating) field filling up the studied domain in the absence of the scattering object (truncated microcavity in our case). Thus, if there is only one (homogeneous) medium alongside the scattering object as in our case, then the background field is simply the incident field $\vec{E}_0$. Conversely, when several dielectric materials are implicated, the background field is assessed for these prior to determining the scattered field.

As a result, the background field in our model reduces to the incident field as follows:

$$\vec{E}_b = (0, E_0 \exp(j.k_0.n_w.z), 0)$$

$E_0 = 1V/m$, being the amplitude of the incident field we set in our model, n_w=1.33 is the refractive index of water medium and z is the coordinate along z-axis. The incoming field is linearly polarized along the y-axis as it can be noticed.

As previously noted, the only dielectric material present in the model is water and is simulated through its refractive index. On the other hand, gold metal is simulated in Comsol using a complex refractive index (n_{Au}=n+jk) so that the dispersive and absorption properties of the metal are taken into account in the frequency range of interest. Specifically, the analytical expressions for the real and imaginary part of the gold refractive index are given by:

$$n = real(n_{Au}) = p_0 + p_1.\lambda_0 + p_2.{\lambda_0}^2 + \frac{p_3}{(p_5 + (\lambda_0 - p_4)^2)}$$

$$k = imag(n_{Au}) = a_0 + a_1.\lambda_0 + a_2.{\lambda_0}^2 + a_3.{\lambda_0}^3$$

where λ is the incident field wavelength in vacuum, The parameters p_0 to p_5 and a_0 to a_3 used to fit the experimental measurements of Johnson and Christy (Johnson and Christy 1972), are:

$$p_0 = -0.143248985972586, p_1 = 0.281900909294717\mu\text{m}^{-1},$$
$$p_2 = 0.107160322951754\mu m^{-2}, p_3 = 0.003300695610667\mu\text{m}^2$$
$$p_4 = 0.458396830236043\mu m, p_5 = 0.002331850748572\mu m^2.$$

$$a_0 = -4.088545252717686, a_1 = 14.563472861595455\mu m^{-1},$$
$$a_2 = -4.814569775061443\mu m^{-2}, a_3 = 0.869581386738358\mu m^{-3}.$$

The wavelength is λ_0 = 593 nm, for convenience for the laser available for future experimental related work. Finally, being only concerned with the stationary solutions (transient phenomena are irrelevant here) we solve equation (1) for $\vec{E}_s$in stationary frequency domain in Comsol.

At this stage – before considering inclusion of the nanopore in the microcavity structure, the model was validated by comparing the field distribution of some numerically simulated data with some analytical results (R A Yadav 2004) that yield resonance modes of a dielectric sphere (water sphere) embedded in a perfect metal (good approximation in visible range), and compared field distribution of these modes with their counterparts obtained numerically. Figure S1 contains the data for the normalized electric field distribution in a water filled cavity surrounded by gold for an incident wavelength of λ_0 = 0.637μm obtained by the analytical calculation (fig. S1a) or FEM simulation (fig. S1b).

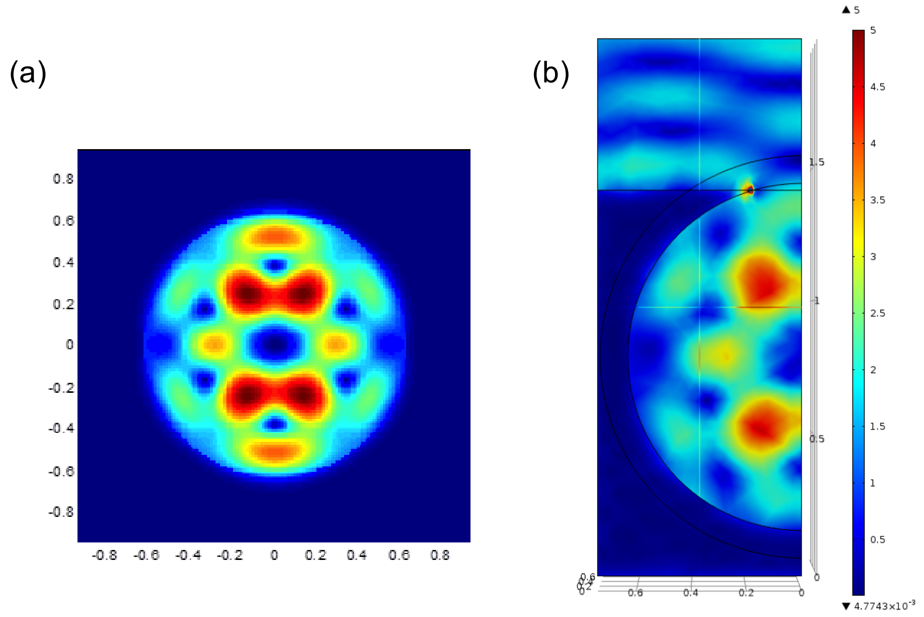

Figure S1: (a) Normalised electric field distribution in the water filled microcavity embedded in perfect metal (gold), obtained analytically. (b) Same as in (a) but by using FEM simulation.

The FEM simulation (fig. S1b) has been performed only for a quarter of the cavity taking advantage of the two symmetry planes of the system. One clearly observes the good match between field distributions achieved by numerical (fig. S1b) or analytical means (fig. S1a).

Prior to studying the interaction between the microcavity with an integrated nanopore, we first examined the normalized electric field distribution of an isolated pore (rectangular nanopore) in a thick gold metal film (same boundary conditions as for the whole model used for the FEM simulations). In figure S2 and S3, we present the field distribution of a square pore whose dimensions are: 50nm for the square sides, and 150nm for the depth.

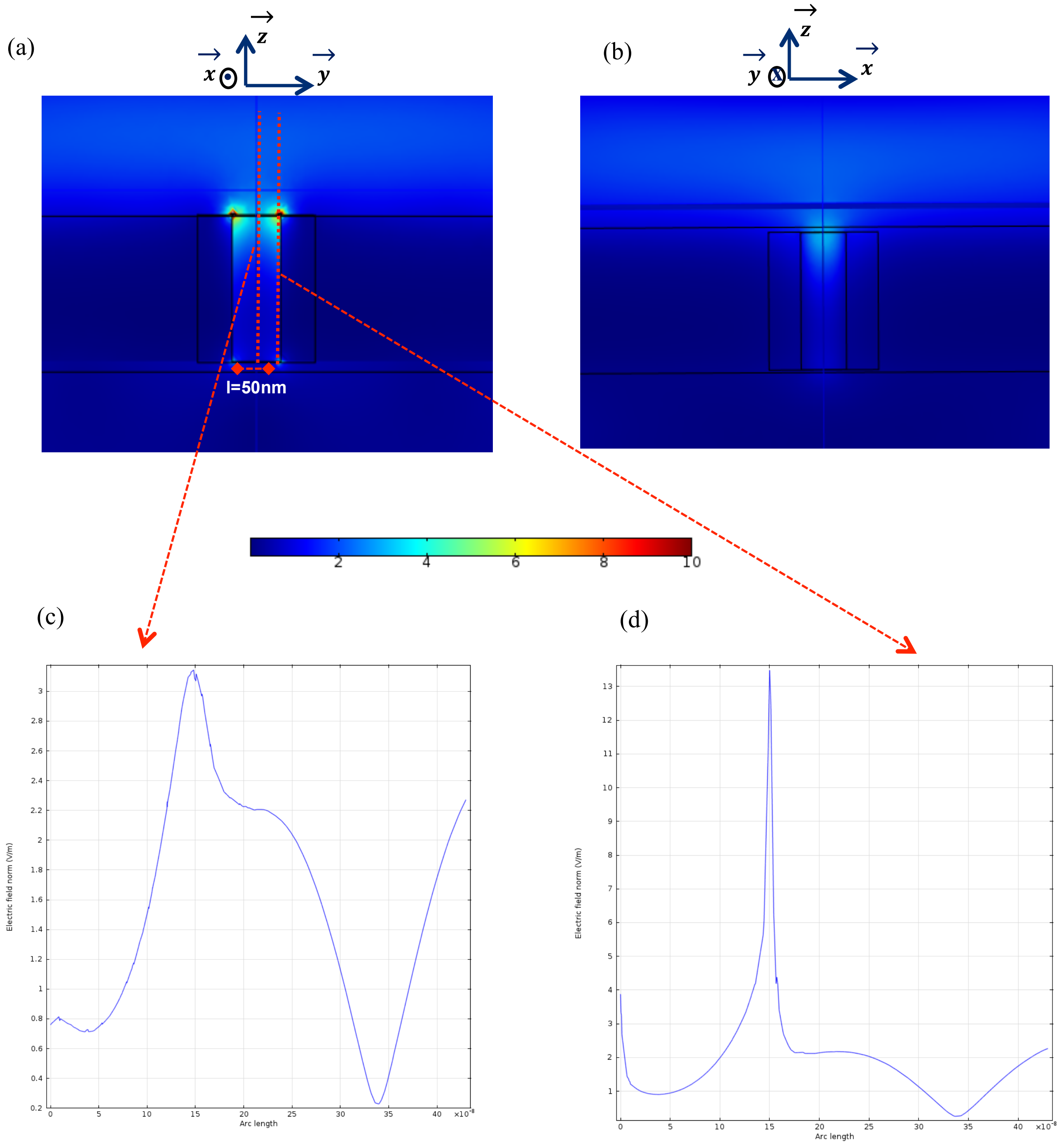

Figure S2: (a) Normalised electric field distribution in a square pore (50nm square and 150nm deep) filled with water (in *ozy* plane), and excited with a normally incident plane wave of wavelength in vacuum λ_0=0.637μm. (b) Same as in (a) but in *ozx* plane. (c) Plot of the normalised electric field along the *z*-axis (located in the middle of the pore as shown in figure (a)). (d) Same as in (c) but along the dashed line in the *z*-axis near to the pore edge – shown in (a). The colour scale bar is the normalized field with respect to the electric field of the incident plane wave. In figure S3, is a top view of the electric field (normalised distribution) within the pore.

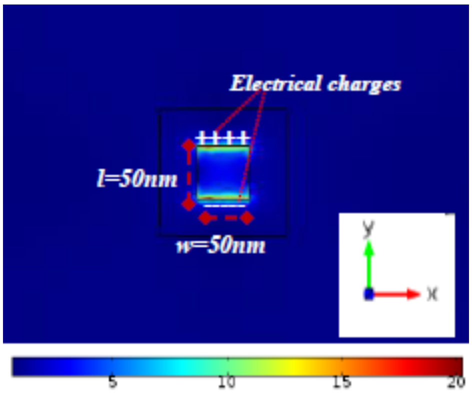

Figure S3: Top view of the electric field (normalised distribution) within the pore (Figure S2(a)).

The gold microcavity (1.2μm diameter, depth 0.6μm) with a square nanopore (50nm square) centrally located in the base of a microcavity are shown in figure S4 (Note: square nanopore with no rounded corners).

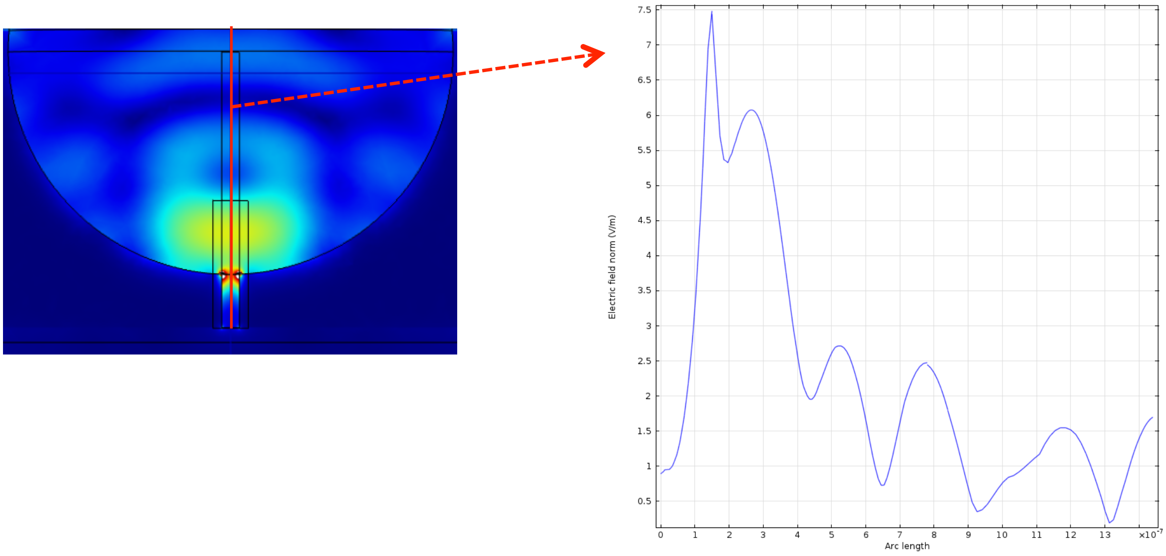

Figure S4: (a) Electric field normalized distribution (in *ozy* plane) in a truncated water cavity (of radius 0.63μm and truncation degree t=0.54) coupled to square pore of dimensions, 50nm×50nm×150nm deep (filled with water). The system is excited with a normally incident plane wave of wavelength λ_0=0.637μm. (b) Electric field normalized profile along an ascendant axis (shown as a red solid line in figure S4(a).

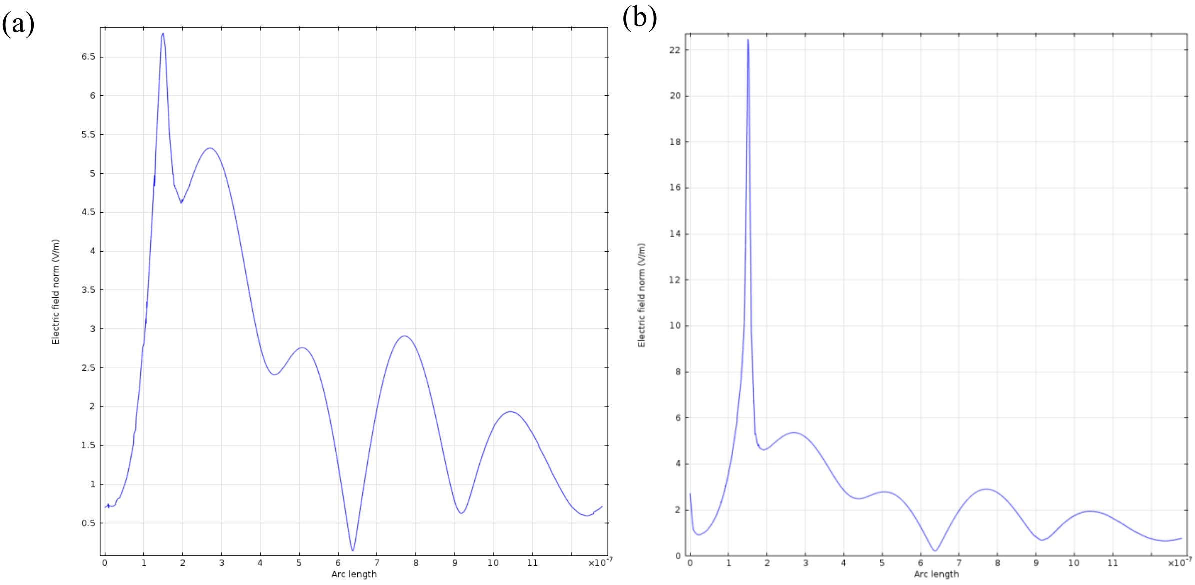

Figure S5 (a) Electric field normalized profile along an ascendant arc that is situated in the centre/middle of the pore and cavity (See figure 1(b)). (b) Same as in (a) but very near to one of the pore edges (that sustains the SPP resonance) (See Figure 4 in the paper (red dashed lines for each arc)).

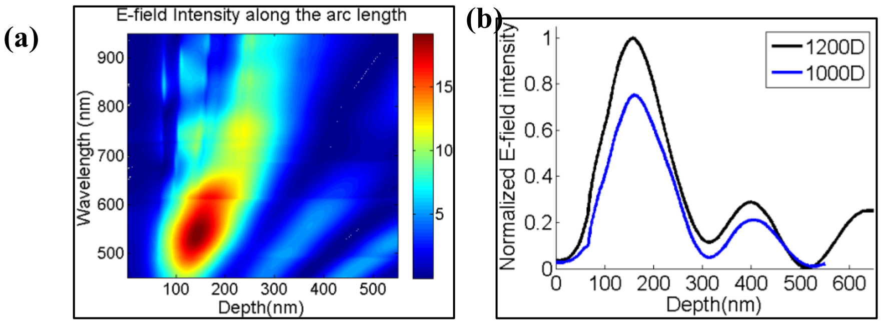

Figure S6 Electric field response from 1000nm sphere (a) field distribution map (b) comparison of normalized field intensity between 1000nm and 1200nm sphere at the operation wavelength of 595nm

Fabrication supplementary information:

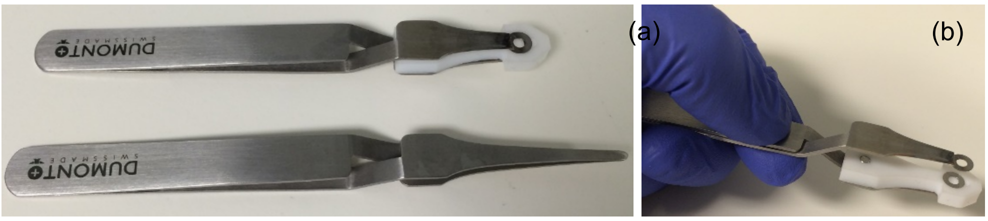

Figure S7 (a) Photograph of DUMONT crossover tweezer as supplied by Agar scientific (bottom) and modified tweezer to hold the free standing gold membrane together with the TEM grid (top). (b) The photograph illustrates the open device used to hold and press together the gold membrane and TEM grid before washing away the epoxy layer.

Electroplating method optimization to create microcavities that confirm to the polymer sphere assembles

Gold electroplating was performed using a three-electrode configuration (Figure S8) in which the work piece to be plated is the working electrode (WE) while the control electrode (CE) completes the electrical circuit and the third electrode is the reference electrode (RE) referred as an arbitrary zero point of electrode potential to detect the electrical potential difference between WE and RE and hence control the charge flow through the cell. Here the working electrode is the 100nm gold substrate with self-assembled 1200nm polystyrene spheres which are used as a template, the counter electrode is large area (~1.1cm^2) platinum wire mesh gauze and the reference electrode is a saturated calomel reference electrode. To build an electrochemical cell, a glass beaker is cleaned by rinsing thoroughly in deionised water and thoroughly drying with clean-room tissue and drying in an oven. The counter electrode is cleaned by rinsing in a stream of deionised water followed by either holding in a butane/propane fire torch or baking with a hot plate at 40˚C for a few seconds until dry. The reference electrode that was usually stored in saturated potassium chloride (KCl) solution, is cleaned by rinsing under a stream of deionised water and drying with a clean room tissue. The drying step is very important as the gold plating solution becomes unstable if in contact with water. The electrodes are arranged inside the cell so that the counter and working electrode are opposite each other (Figure S8(b)) and the reference electrode is held near the working electrode, but does not obstruct the sample area. The beaker is filled with gold plating solution so that it covers the deposition area of spheres on the working electrode completely, but does not cover the gold coated area used for electrical contact with the potentiostat (Figure S8(a)). For this gold electroplating method, we used gold sulphite, mild alkaline solution with pH=9 Gold ECF 60, brightener E3 containing 10g/dm^3 gold from Metalor and Autolab PG-STAT12 with GPES 4.9 software.

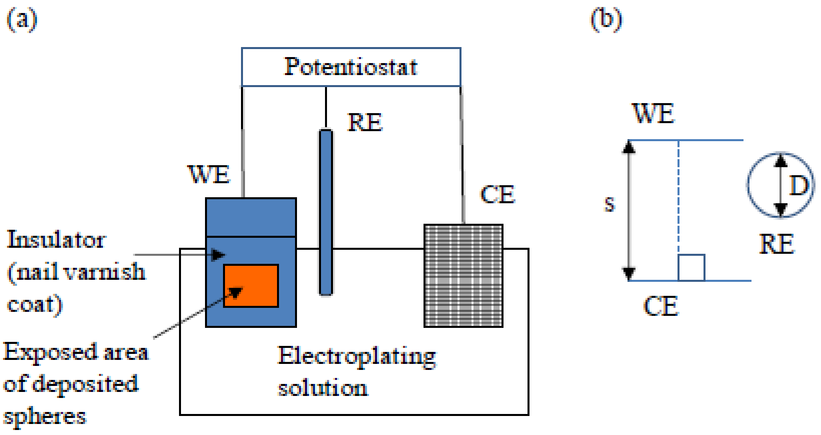

Figure S8: Three-electrode electrochemical cell setup (a) components (b) top view of optimum relative position of electrodes; CE=counter electrode (platinum wire mesh, area is about 1cm x 1cm), WE=working electrode, RE=reference electrode (saturated calomel reference electrode, diameter (D) is equal to about 0.6 cm, the separation (s) between WE and CE is about 1 cm.

Two parameters need to be controlled during film deposition (i) film thickness and (ii) deposition speed. The film thickness (δ) is controlled by the total charge used in the deposition such as, $\square \propto \frac{Q}{A}$ where Q is the total

charge to the working electrode and equals, $Q = \int Idt$ where I is the current and t is the time and A is deposition area. According to Faraday's Law, the deposition thickness, δ(cm) can be solved by,

$$\delta = \frac{W}{nF\rho A}\int Idt \quad (1)$$

where W is the atomic weight (g/mol), n is the number of electrons take part in the reduction, F is the Faraday constant (~9.65x10^4 C/mol), ρ is the density of the metal (g/cm^3) and A is the area of deposition (cm^2) (Yinlun and Helen 2007). Therefore, one can calculate the total charge to be flowed for the required thickness for the sample from the equation:

$$Q(C) = 96500\frac{\rho}{0.8\times10^7 W}xyt[1-\frac{1}{2\sqrt{3}}\frac{t}{R^2}(3R-t)] \quad (2)$$

where W is the atomic weight, 197.0 grams/mole in the case of gold, ρ (g/cm^3) is the density of the metal, 19.32 g/cm^3 in the case of gold, t (nm) is the film thickness, R (nm) is the sphere radius, and x*y (cm^2) is the rectangular deposition area A. However, there is reliable way to control the film thickness via the software. Normally, the film deposition over the nanometer sphere substrate was carried out using the electrode potential at around -0.72V to -0.75V with respect to the reference electrode for the gold metal and at -0.9V for the silver. By monitoring the current versus time curve (Figure S9) with the software. It is easy to control the thickness of the resulting film. During the electrodeposition of gold around the assembled sphere template, sequential oscillations are observed with a decrease of current applied. At the beginning of the deposition each sphere of the template is in contact with the electrode surface at only one point resulting in both electrode surface and the deposition current are maximum as shown in figure S9 –for the first curve- the gold is just starting to be electrodeposited (25% of depth). Then the gold electroplates around the sphere, the active electrode surface area decreases continuously and passes through a lowest minimum when the film is deposited to 50% of the depth of the sphere (figure S9-red curve) so that the metal thickness reaches the middle of the monolayer of spheres. After that the current will increase again due to the increase of the active electrode area (figure S9 – blue curve and green curve) (Ismail 2010). But it must be noted: a rapid decrease of the current can cause a reduction in the film quality such as holes, roughness and in worst case, the film will not deposit around the full curvature of the sphere. In order to control the smoothness of the deposited film, the brightener is added to the gold plating solution in which 400μL of brightener is added to 30mL of plating solution. Using this mixture of solution, the film is possible to electroplate with the depth of 50% of the 1200nm sphere so that the fabrication process stop point is detected when the current passes through the minimum point.

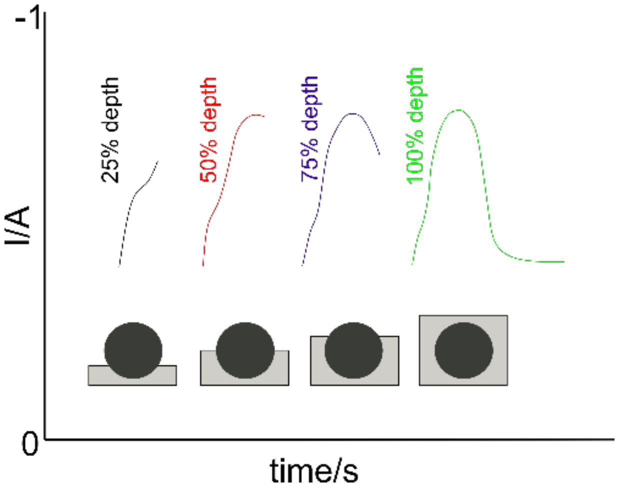

Figure S9 Current versus time curves for gold electroplating of polymer micro-sphere templates shows that the current controls the film thickness (Ismail 2010)

First, the deposition was tried with the standard electrode potential -0.72V versus reference electrode for gold. From the SEM image and current versus time curve (figure S10), it can observe that the plated gold did not conform to the sphere and is not present at the bottom of the sphere because the deposition speed is too rapid.

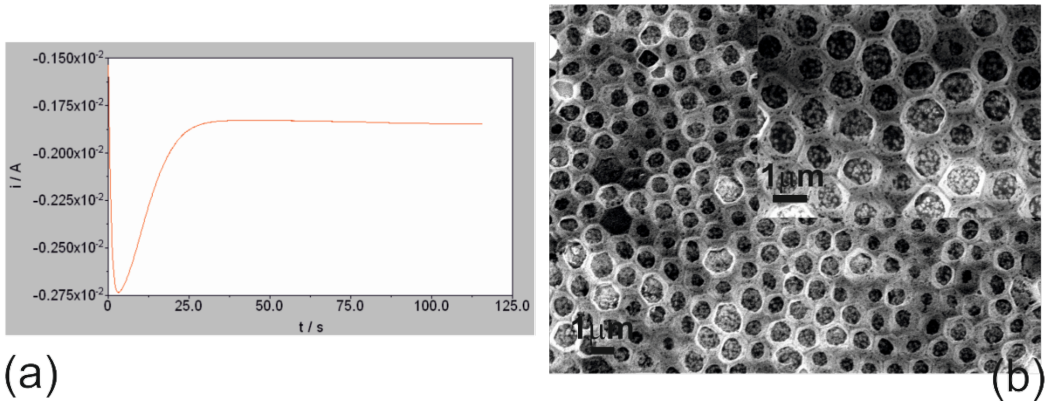

Figure S10 (a) Current versus time during deposition, (b) SEM image of the resulting film.

Therefore, the current versus potential curve is analyzed for the estimated deposited area during the scan by cyclic voltammetry. The current curve under the reverse bias condition has to be evaluated because a more negative bias and hence more negative current can cause the deposition of the metallic gold to be too rapid. Therefore from this current versus potential curve, a suitable potential can be selected to control the deposition speed. Here we tried different minimum potentials to control the deposition speed and conclude that the potential just before the current goes to zero is the optimum potential to achieve the deposition over the full curvature of the sphere. Figure S11 (a) and (b) show the cyclic voltammetry scans for the deposited area of 0.5cm^2 and 1cm^2 respectively. From the curves, the electrode potential of -0.63V is selected for 1st sample (figure S11(a)) which is below the shoulder of the reverse bias curve which is far from the zero value of the current. And the electrode potential, -0.6V is selected for 2nd sample (figure S11(b)) that is just over the shoulder of the reverse bias curve which is close to the aforementioned optimum point.

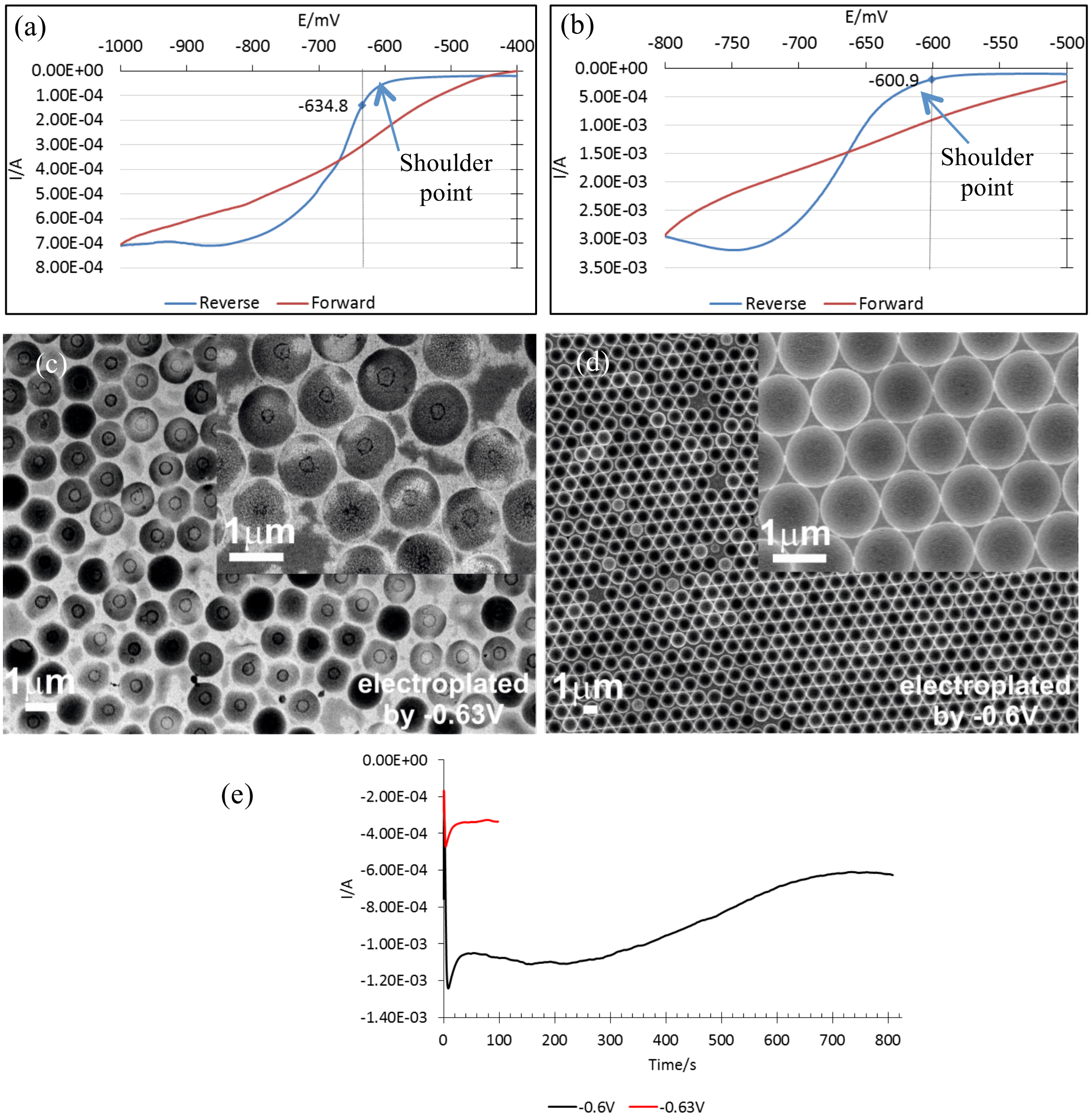

Figure S11 (a) Current versus potential curve for the deposited area, of (a) $0.5cm^2$, and (b) $1cm^2$, (Note: blue diamond symbol and solid lines in (a) and (b) are guides to the selected electrode potential). (c) SEM image for the deposited film electroplated with an applied potential of (c)-0.63V, and (d) SEM image for the deposited film electroplated (-0.6V). (Inserts are magnified views) (e) Current versus time curve for the same film thickness electroplated by different potential (-0.6V (black) and -0.63V (red))

The deposited film electroplated by -0.63V vs. reference electrode gives the better film quality compared to the resulting film electroplated by standard potential -0.72V. However small defects can be seen from the SEM image that shows the lack of gold deposition at the bottom of the sphere. The deposited film electroplated by -0.6V achieves the optimum deposition over the full curvature of the sphere with a surface that conforms to the sphere surface profile. The deposition speed can be seen in figure S11(e) which shows the different deposition speeds for the same film thickness, 600nm. The film thickness is achieved after 800seconds with the potential -0.6V while the desired film thickness is reached within 100 seconds applying the potential -0.63V.

<u>Supplementary Information in the form of videos</u>

Video 1: (video1.mp4) The calculated *E*-field intensity over the cross-section of the nanoporous micro-cavity for different excitation wavelengths. The cross-section was taken along the XZ plane at the center of the gold membrane, as shown in figure 1(b) in the paper. The nanopore shape was fixed to 50nm x 50nm square and the diameter of sphere was 1200nm and the height of the cavity was 600nm. The localized *E*-field was highly confined at just above the nanopore in the fluorescent regime 450nm-700nm wavelengths. The e-field spreads along the curve of the cavity with wavelength up to 650nm excitation wavelength, and gradually disperses over the cavity in the near infrared regime.

References

Ismail, I.M.I., 2010. Synthesis of nanostructured films from template electrodeposition technique. Journal of Saudi Chemical Society 14(4), 351-356.

Johnson, P.B., Christy, R.W., 1972. Optical Constants of the Noble Metals. Physical Review B 6(12), 4370-4379.

R A Yadav, I.D.S., 2004. <Normal modes and quality factors of shperical dielectric resonantors_I_Shielded dielectric sphere.pdf>. PRAMANA journal of physics 62(6), 17.

Yinlun, H., Helen, H.L., 2007. Electroplating. Encyclopedia of Chemical Processing, pp. 839-848. Taylor & Francis.
